# A Novel Insight Into Fecal Occult Blood Test for the Management of Gastric Cancer: Complication, Survival, and Chemotherapy Benefit After R0 Resection

**DOI:** 10.3389/fonc.2020.526746

**Published:** 2021-02-11

**Authors:** Jun Lu, Binbin Xu, Yu Xu, Yuan Wu, Jianwei Xie, Jiabin Wang, Jianxian Lin, Qiyue Chen, Longlong Cao, Chaohui Zheng, Changming Huang, Ping Li

**Affiliations:** ^1^ Department of Gastric Surgery, Fujian Medical University Union Hospital, Fuzhou, China; ^2^ Department of General Surgery, Fujian Medical University Union Hospital, Fuzhou, China; ^3^ Key Laboratory of Ministry of Education of Gastrointestinal Cancer, Fujian Medical University, Fuzhou, China; ^4^ Department of Pathology, the School of Basic Medical Sciences, Fujian Medical University, Fuzhou, China

**Keywords:** gastric cancer, fecal occult blood test, adjuvant chemotherapy, prognosis, tumor immune microenvironment

## Abstract

**Background:**

Previous studies have shown that the all-cause mortality and non-colorectal cancer mortality of patients with fecal occult blood test (FOBT) positivity are significantly increased, implying that FOBT results may have more prognostic value.

**Methods:**

Retrospective analysis was performed for gastric cancer (GC) patients who underwent R0 gastrectomy from July 2007 to July 2014 at our hospital. Propensity score matching (PSM) was used to reduce confounding bias and a computerized technique for the nearest available score matching without replacement was applied. The cumulative survival rate was calculated using the Kaplan-Meier method and a log-rank test. Cox proportional hazards regression and logistic regression was used to determine the independent prognostic factors associated with survival and postoperative complications, respectively. The expression level of tumor-associated macrophages (TAMs) and proinflammatory cytokines (TNF-α, IL-6) were evaluated by immunohistochemical (IHC).

**Results:**

A total of 3,003 patients were included and 246 patients (8.2%) were in preoperative FOBT positive status. There was no significant difference in demographic data between preoperative FOBT positive and negative group after a 1:4 PSM. The overall postoperative complications, major complications, and anastomotic leakage were significantly higher in the preoperative FOBT-positive group than in the preoperative FOBT-negative group. Moreover, preoperative FOBT-positivity was an independent risk factor for 5-year overall survival (OS) (HR: 1.32, p = 0.005). For stage II/III patients, the postoperative adjuvant chemotherapy (PAC) benefit was found in preoperative FOBT-negative group (5-year OS: 49.9 *vs*. 36.8%, p = 0.001), whereas the PAC benefit was lost in preoperative FOBT-positive groups (5-year OS: 40.8 *vs*. 37.7% p = 0.896). Finally, IHC found that preoperative FOBT-positivity in patients was significantly associated with higher TAMs infiltration and higher expression of IL-6 and TNF-α in tumor tissues than in the preoperative FOBT-negative group.

**Conclusion:**

As a simple and low-cost method, preoperative FOBT results can predict both complications and survival after R0 gastrectomy for GC. More importantly, stage II/III GC patients with FOBT-positive seem not benefit from PAC alone. Further exploration is warranted.

## Introduction

In the past few decades, despite significant advances in early diagnosis, radical surgery, and chemotherapy, gastric cancer (GC) remains the fifth-most common malignancy in the world and ranks third in tumor-related mortality (1). Radical gastrectomy is the dominant treatment for patients with resectable gastric cancer. Looking for indicators that can effectively predict complications and prognosis in GC patients may help develop individualized treatment options to improve patients’ outcomes.

In addition, there are large differences in the postoperative adjuvant chemotherapy (PAC) response. Increasing numbers of scholars have explored the predictive indicators of chemotherapy response. It has been found that some prognostic scores based on the tumor immune microenvironment (TIM) or gene expression predicted the PAC response in stage II/III GC patients (2–7). However, they have not been routinely used in clinical practice due to their complicated operation and high cost.

The fecal occult blood test (FOBT) has been widely used for screening colorectal cancer (8). Recently, several studies have shown that people with FOBT-positivity have a significantly higher mortality rate, including colorectal and non-colorectal cancer mortality, than those with FOBT-negativity (9, 10), suggesting that FOBT results can become potential population prognostic indicators, especially for cancer patients. However, there are no studies reporting the value of the FOBT in predicting the short-term and long-term effects of R0 after GC patients and the benefits of PAC.

Therefore, the present study aimed to explore the impact of the FOBT results on the long-term prognosis of and PAC benefit in GC patients after radical resection. In addition, we analyzed the relationship between FOBT status and the local tumor immune microenvironment (TIM) by immunohistochemistry (IHC) analysis to speculate the potential molecular mechanism underlying how the FOBT results affects clinical efficacy in the treatment of GC.

## Materials and Methods

### Study Population

In this retrospective analysis, data were collected from 3,343 patients diagnosed with primary gastric adenocarcinoma at Department of Gastric Surgery, Fujian Medical University Union Hospital (FMUUH) from July 2007 to July 2014. Two attending physicians staged the tumor before the operation according to gastroscopy, total abdominal CT and enhancement, total abdominal ultrasonography, and other examination results (11). Digital rectal examination and total abdominal CT were performed to preliminarily determine whether the patient has a colorectal tumor. Colonoscopy is performed only when the patient is suspected of having a colorectal tumor by total abdominal CT. The exclusion criteria were as follows: 1) patients with a history of other malignant tumors within 5 years (n = 18); 2) neoadjuvant chemotherapy (n = 89); 3) intraperitoneal or distant metastasis confirmed during or after the operation (n = 103); 4) gastric stump cancer (n = 99); and 5) unavailable data of the FOBT results (n = 31). A total of 340 patients were excluded. The remaining 3003 patients undergoing radical gastrectomy were entered into the statistical analysis (see flowchart in **Supplementary Figure S1**). Postoperative pathological TNM (pTNM) staging was based on the 7th American Joint Committee on Cancer (AJCC) staging system. Patients in stage I were excluded from a subset analysis assessing the benefits of PAC. Written informed consent was obtained from all patients before sample collection, and the study procedures were approved by the Institutional Review Board of Fujian Medical University Union Hospital.

### Fecal Occult Blood Test

Preoperative stool routine and FOBT were administered to all patients at our institution, unless patients’ refusal or an inappropriate physical condition. The fecal occult blood test kit was obtained from Baso Diagnostics, China, with a detection limitation of 50 μg (Hb)/g (stool), as previously described (12). The FOBT was usually conducted within 1 week before surgery.

### Immunohistochemistry

Formalin-fixed, paraffin-embedded GC surgical specimens, obtained from 120 GC patients, were used for immunohistochemistry (IHC). The contents of infiltrated macrophages, interleukin-6 (IL-6), and tumor necrosis factor-α (TNF-α) in the individual GC specimens were characterized by IHC using an avidin-biotin peroxidase complex method, as previously reported (13). Briefly, Slides (4-μm thick consecutive paraffin sections) from the blocks with the highest tumor content for each sample were used for immunohistochemical staining and immersed in xylene and rehydrated through graded concentrations of ethanol followed by PBS buffer and deionized water for 5 min each. Slides were then heated to 100°C for 20 min in a pH 9 Tris-based solution. All slides were incubated with the primary antibodies for 60 min at 37°C for 1 h (dilutions: mouse anti-CD68 1:500, Maixin Bio, Fuzhou, China; rabbit anti-human TNF alpha 1:300, Abcam ab6671, Cambridge, UK; mouse anti-human IL-6 1:400, Abcam ab9324, Cambridge, UK) and were then washed. A secondary antibody for mouse IgG was added for 30 min and the slides were again washed. The sections were processed with the universal SP Elivision-Plus Kit (Maixin Bio, Fuzhou, China). Finally, the sections were counterstained with hematoxylin.

### Evaluation of Immunohistochemical Staining

Individual specimens were evaluated by two pathologists (Xu Y and Wu Y) in a blind manner. CD68+ TAMs were estimated by counting the number of CD68+ TAMs in each of the 3 tissue cores from each patient tumor sample, and the mean of 3 counts was recorded. The percentages of CD68+ cells in three representative high power fields of individual samples were analyzed for macrophage infiltration and were scored as 0 (<5% of CD68+cells), 1 (5–25%), 2 (>25–50%), or 3 (>50%), as previously described (14). A score of 0 or 1 with anti-CD68 on immunohistochemistry was regarded as “low TAM infiltration” and 2 or 3 staining as “high TAM infiltration” (14).

IL-6 or TNF-α positivity was scored 0 to 3 as a proportion of tumor cells as follows: <5% = 0; 5% to 25% = 1; 26% to 50% = 2; or >50% = 3. Staining intensity was also scored as negative (0), weakly positive (1), moderately positive (2), or strongly positive (3). Based on the sum of these two scores, patients were then dichotomized into “low expression” (score of 0–2) and “high expression” (score of 3–6) groups (15). (**Supplementary Figures S2**–**S4**).

### Propensity Score Matching

The propensity scores were calculated using a logistic regression model, and the following covariates were included: age, sex, the Charlson Comorbidity Index (CCI), American Society of Anesthesiologists (ASA) grade, adjuvant chemotherapy, pT, pN, hemoglobin (Hb) level, and albumin (Alb) level. We used a computerized technique for the nearest available score matching without replacement (11) using SPSS 18.0 (SPSS Inc., Chicago, IL, USA).

### Complications

Complications were defined as previously described (11). Postoperative complications were graded according to the Clavien-Dindo classification system; complications greater than grade III were defined as serious complications (16).

### Surgery

In the present study, all the patients had undergone D2 radical gastrectomy with the same group of surgeons. The following lymphadenectomy sequences were performed: 1) for distal gastrectomy, no. 6 → no. 7, 9, 11p → no. 3,1 → no. 8a, 12a, 5 → no. 4sb; and for 2) total gastrectomy: no. 6 → no.7, 9, 11p → no. 8a, 12a, 5 → no. 1 → no. 4sb → no. 10,11d → no. 2. The additional details were described in the previous study (17–20).

### Adjuvant Chemotherapy

According to the patient’s wishes and their physical condition, fluoride-based adjuvant chemotherapy was recommended for most patients with pathological stage II and III disease in our center, as previously described (21). Final decision to administer adjuvant chemotherapy was made after careful discussion between the clinician and the patients.

### Follow-Up

The median follow-up time was 72 months (95% CI 71–74 months). Overall survival (OS) was defined as the period from the date of surgery to the date of death or the final follow-up (11). Postoperative follow-ups were performed every 3 months for 2 years and then every 6 months from years 3 to 5. Most routine patient follow-up appointments included a physical examination, laboratory tests, chest radiography, abdominal ultrasonography or CT and an annual endoscopic examination (11).

### Statistical Analysis

Continuous variables are reported as the means ± SD. Categorical and continuous variables were compared using a χ2 test or Fisher’s exact test and a t test, respectively. The cumulative survival rate was calculated using the Kaplan-Meier method and a log-rank test. A Cox proportional hazards regression model was used to determine the independent prognostic factors associated with survival. All statistical analyses were performed using SPSS v.18.0 for Windows (SPSS Inc., Chicago, IL, USA) and R (https://www.r-project.org/). Values of p lower than 0.05 were considered statistically significant.

## Results

### Clinicopathological Characteristics of the Patients

**Table 1** shows the demographic data of all the patients (n = 3,003) and the propensity score-matched patients (n = 1,230). A total of 246 patients (8.2%) were FOBT-positive with a worse preoperative status (e.g., age, ASA score, CCI, Hb, Alb), and the tumor stage was more advanced (all p<0.05). In the FOBT (+) group, patients with the atrial fibrillation (1.2%), coronary heart disease (3.3%), peptic ulcer (1.2%), or inflammatory bowel disease (0%) only occupied a smaller proportion. After propensity score matching (PSM) for 1:4 to eliminate the baseline bias, no significant differences between the FOBT-positive group (n = 246) and the FOBT-negative group (n = 984) were observed in clinicopathological characteristics (all p>0.05).

**Table 1:** Clinicopathological characteristics of patients before and after matching.

	All patients			Propensity-matched patients		
	FOBT (−) (n = 2,757)	FOBT (+) (n = 246)	p value	FOBT (−) (n = 984)	FOBT (+) (n = 246)	p value
Age			<0.001			0.819
<65	1,734 (62.9%)	127 (51.6%)		516 (52.4%)	127 (51.6%)	
≥65	1,023 (37.1%)	119 (48.4%)		468 (47.6%)	119 (48.4%)	
Sex n (%)			0.026			0.526
Female	704 (25.5%)	47 (19.1%)		206 (20.9%)	47 (19.1%)	
Male	2,053 (74.5%)	199 (80.9%)		778 (79.1%)	199 (80.9%)	
Charlson Comorbidity Index, n (%)			0.005			0.509
0	1,908 (69.2%)	154 (62.6%)		607 (61.7%)	154 (62.6%)	
1	628 (22.8%)	58 (23.6%)		261 (26.5%)	58 (23.6%)	
≥2	221 (8.0%)	34 (13.8%)		116 (11.8%)	34 (13.8%)	
Comorbidity, n (%)						
Atrial fibrillation	14 (0.5%)	3 (1.2%)	0.158	5 (0.5%)	3 (1.2%)	0.369
Coronary heart disease	107 (3.9%)	8 (3.3%)	0.167	52 (5.3%)	8 (3.3%)	0.186
Peptic ulcer	21 (0.8%)	3 (1.2%)	0.441	6 (0.5%)	3 (1.2%)	0.369
IBD	1 (0.04%)	1 (0.4%)	0.157	0 (0.0%)	1 (0.4%)	0.200
ASA			0.020			1.000
<3	2,626 (95.2%)	226 (91.9%)		904 (91.9%)	226 (91.9%)	
≥3	131 (4.8%)	20 (8.1%)		80 (8.1%)	20 (8.1%)	
BMI			0.428			0.774
<25	2,335 (84.7%)	213 (86.6%)		845 (85.9%)	213 (86.6%)	
≥25	422 (15.3%)	33 (13.4%)		139 (14.1%)	33 (13.4%)	
Tumor size n (%)			0.209			0.253
<50 mm	1,471 (53.4%)	121 (49.2%)		524 (53.3%)	121 (49.2%)	
≥50 mm	1,286 (46.6%)	125 (50.8%)		460 (46.7%)	125 (50.8%)	
Tumor location n (%)			0.568			0.744
Upper	723 (26.2%)	62 (25.2%)		281 (28.6%)	62 (25.2%)	
Middle	552 (20.0%)	49 (19.9%)		188 (19.1%)	49 (19.9%)	
Lower	1,182 (42.9%)	101 (41.1%)		393 (39.9%)	101 (41.1%)	
Mix	300 (10.9%)	34 (13.8%)		122 (12.4%)	34 (13.8%)	
Gastrectomy extent n (%)			0.350			0.350
Distal	1,437 (52.1%)	139 (56.5%)		1,437 (52.1%)	139 (56.5%)	
Total	1,263 (45.8%)	101 (41.1%)		1,263 (45.8%)	101 (41.1%)	
Others	57 (2.1%)	6 (2.4%)		57 (2.1%)	6 (2.4%)	
Reconstruction			0.031			0.596
B-I	938 (34.0%)	75 (30.5%)		307 (31.2%)	75 (30.5%)	
B-II	297 (10.8%)	18 (7.3%)		83 (8.4%)	18 (7.3%)	
Roux-en-Y	1,437 (52.1%)	139 (56.5%)		556 (56.5%)	139 (56.5%)	
Others	85 (3.1%)	14 (5.7%)		38 (3.9%)	14 (5.7%)	
Histologic type n (%)			0.976			0.863
Well	136 (4.9%)	12 (4.9%)		41 (4.2%)	12 (4.9%)	
Moderate	982 (35.6%)	86 (35.0%)		339 (34.5%)	86 (35.0%)	
Poor	1,639 (59.4%)	148 (60.2%)		553 (56.2%)	148 (60.2%)	
Lymphovascular invasion n (%)			0.314			0.741
Absent	2,014 (73.1%)	187 (76.0%)		738 (75.0%)	187 (76.0%)	
Present	743 (26.9%)	59 (24.0%)		246 (25.0%)	59 (24.0%)	
Adjuvant chemotherapy n (%)			<0.001			0.819
Absent	1,596 (57.9%)	111 (45.1%)		452 (45.9%)	111 (45.1%)	
Present	1,161 (42.1%)	135 (54.9%)		532 (54.1%)	135 (54.9%)	
pT stage n (%)			<0.001			0.253
T1	691 (25.1%)	41 (16.7%)		142 (14.4%)	41 (16.7%)	
T2	337 (12.2%)	30 (12.2%)		103 (10.5%)	30 (12.2%)	
T3	759 (27.5%)	55 (22.4%)		280 (28.5%)	55 (22.4%)	
T4	970 (35.2%)	120 (48.8%)		459 (46.6%)	120 (48.8%)	
pN stage n (%)			<0.001			0.917
N0	1,060 (38.4%)	67 (27.2%)		252 (25.6%)	67 (27.2%)	
N1	414 (15.0%)	36 (14.6%)		138 (14.0%)	36 (14.6%)	
N2	444 (16.1%)	43 (17.5%)		171 (17.4%)	43 (17.5%)	
N3	839 (30.4%)	100 (40.7%)		423 (43.0%)	100 (40.7%)	
pTNM stage n (%)			<0.001			0.861
I	830 (30.1%)	49 (19.9%)		184 (18.7%)	49 (19.9%)	
II	618 (22.4%)	49 (19.9%)		190 (19.3%)	49 (19.9%)	
III	1,309 (47.5%)	148 (60.2%)		610 (62.0%)	148 (60.2%)	
Hemoglobin n (%)			<0.001			0.423
<90 g/L	276 (10.0%)	69 (28.0%)		232 (23.6%)	69 (28.0%)	
≥90 g/L	2,481 (90.0%)	177 (72.0%)		752 (76.4%)	177 (72.0%)	
Albumin n (%)			<0.001			0.529
<35 g/L	585 (21.2%)	116 (47.2%)		442 (44.9%)	116 (47.2%)	
≥35 g/L	2,172 (78.8%)	130 (52.8%)		542 (55.1%)	130 (52.8%)	
Death n (%)			<0.001			0.001
no	1,716 (62.2%)	111 (45.1%)		561 (57.0%)	111 (45.1%)	
yes	1,041 (37.8%)	135 (54.9%)		423 (43.0%)	135 (54.9%)	

FOBT indicates fecal occult blood test; ASA, American Society of Anesthesiologists; BMI, body mass index; IBD, inflammatory bowel disease.

### Perioperative Outcome After Surgery

The mean operative time and intraoperative blood loss were comparable between the FOBT-positive group and the FOBT-negative group (216.58 ± 72.72 min *vs*. 213.82 ± 72.24 min, p = 0.592; 124.22 ± 177.43 ml *vs*. 117.04 ± 132.84 ml, p = 0.483, respectively). In addition, from the perspective of postoperative recovery, there were no significant differences in the time to flatus (3.85 ± 1.35 days *vs*. 3.72 ± 1.22 days, p = 0.099), the time to food intake (5.12 ± 1.99 days *vs*. 4.90 ± 1.61 days, p = 0.463) and the postoperative hospital stays (15.47 ± 8.32 days *vs*. 15.02 ± 8.66 days, p = 0.150) (**Table 2**).

**Table 2:** Perioperative outcome and postoperative complication after surgery.

	FOBT (−) (n = 984)	FOBT (+) (n = 246)	p value
Overall complication	146 (14.8%)	50 (20.3%)	0.035
Bleeding	12 (1.2%)	5 (2.0%)	0.357
Digestive tract fistula	10 (1.0%)	9 (3.7%)	0.003
Ileus	6 (0.6%)	2 (0.8%)	1.000
Wound infection	22 (2.2%)	8 (3.3%)	0.355
Abdominal infection	23 (2.3%)	8 (3.3%)	0.413
Pneumonia	76 (7.7%)	21 (8.5%)	0.672
Cardiovascular system	6 (0.6%)	2 (0.8%)	1.000
Liver system	3 (0.3%)	2 (0.8%)	0.590
Urinary system	4 (0.1%)	2 (0.8%)	0.606
Clavien-Dindo classification			0.047
< 3	951 (96.6%)	231 (93.9%)	
≥ 3	33 (3.4%)	15 (6.1%)	
Time to postoperative complication (day)	5.66 ± 3.44	6.60 ± 4.80	0.205
Operative time (min)	213.82 ± 72.24	216.58 ± 72.72	0.592
Intraoperative blood loss (ml)	117.04 ± 132.84	124.22 ± 177.43	0.483
Postoperative hospital stay (day)	15.02 ± 8.66	15.47 ± 8.32	0.150
Time to flatus (day)	3.72 ± 1.22	3.85 ± 1.35	0.099
Time to food intake (day)	4.90 ± 1.61	5.12 ± 1.99	0.463

FOBT indicates fecal occult blood test.

### Postoperative Complications

Overall, postoperative complications occurred in 50 (20.3%) and 146 (14.8%) patients in the FOBT-positive and FOBT-negative groups (p = 0.035), respectively. Among the surgical complications, the incidence of anastomotic fistula was significantly higher in the FOBT-positive group than in the FOBT-negative group [9 (3.7%) *vs*. 10 (1.0%), p = 0.003]. Postoperative ileus, abdominal bleeding, wound infection and abdominal infection were not significantly different between the two groups. Among the non-surgical complications, there were no significant differences in postoperative pneumonia or disorders of the cardiovascular, liver, and urinary systems between the two groups. According to the Clavien-Dindo classification, the incidence of major complications in the FOBT-positive group was significantly higher than that in the FOBT-negative group [15 (6.1%) *vs*. 33 (3.4%), p = 0.047]. In terms of postoperative complications, there was no significant difference in the time of postoperative complications between the FOBT-positive patients and negative patients (6.60 ± 4.80 days *vs*. 5.66 ± 3.44 days, respectively, p = 0.205).

### Univariable and Multivariate Analyses of Factors Associated With Overall Survival

In the univariate analysis, age≥65, BMI≥25, FOBT-positive, histologic type, tumor location, lymphovascular invasion, pTNM stage, preoperative Hb<90 g/L, and preoperative Alb<35 g/L were closely related to overall survival, all p<0.05. In the multivariate analysis, age≥65, FOBT-positive, pTNM stage, and preoperative Alb<35 g/L were independent prognostic factors affecting long-term survival, all p <0.05 (**Table 3**).

**Table 3:** Univariate and multivariate analyses of factors associated with overall survival.

	Univariate analysis			Multivariate analysis		
	HR	95% CI	p value	HR	95% CI	p value
Age ≥ 65 *vs*. < 65	1.543	1.305–1.824	<0.001	1.367	1.153–1.620	<0.001
Male sex *vs*. female	1.021	0.833–1.252	0.843			
Charlson comorbidity index						
0	1.000					
1	0.922	0.756–1.125	0.424			
≥ 2	1.243	0.976–1.582	0.077			
ASA ≥ 3 *vs*. <3	1.149	0.864–1.529	0.340			
BMI ≥25 *vs*. <25	0.720	0.555–0.934	0.013	0.840	0.647–1.091	0.191
FOBT positive *vs*. negative	1.305	1.075–1.584	0.007	1.320	1.085–1.606	0.005
Tumor size ≥ 50 *vs*. <50 mm	1.027	0.870–1.213	0.751			
Tumor location						
Lower	1.000			1.000		
Middle	1.180	0.933–1.493	0.166	1.036	0.818–1.314	0.769
Upper	1.229	0.999–1.512	0.051	1.141	0.926–1.406	0.215
Mix	1.682	1.311–2.158	<0.001	1.226	0.952–1.582	0.114
Histologic type						
Differentiated	1.000			1.000		
Undifferentiated	1.334	1.126–1.581	0.001	1.088	0.914–1.295	0.344
Lymphovascular invasion						
Absent	1.000			1.000		
Present	1.391	1.167–1.658	<0.001	1.077	0.899–1.289	0.423
pTNM stage						
I	1.000			1.000		
II	3.413	2.137–5.449	<0.001	2.894	1.807–4.635	<0.001
III	9.145	6.103–13.908	<0.001	7.645	4.998–11.694	<0.001
Hemoglobin ≥90 *vs*. < 90 g/L	0.684	0.570–0.822	<0.001	0.899	0.739–1.094	0.288
Albumin ≥35 *vs*. < 35 g/L	0.522	0.442–0.618	<0.001	0.663	0.553–0.795	<0.001

FOBT indicates fecal occult blood test; ASA, American Society of Anesthesiologists; BMI, body mass index.

### Overall Survival and Subgroup Analysis

**Figure 1:**
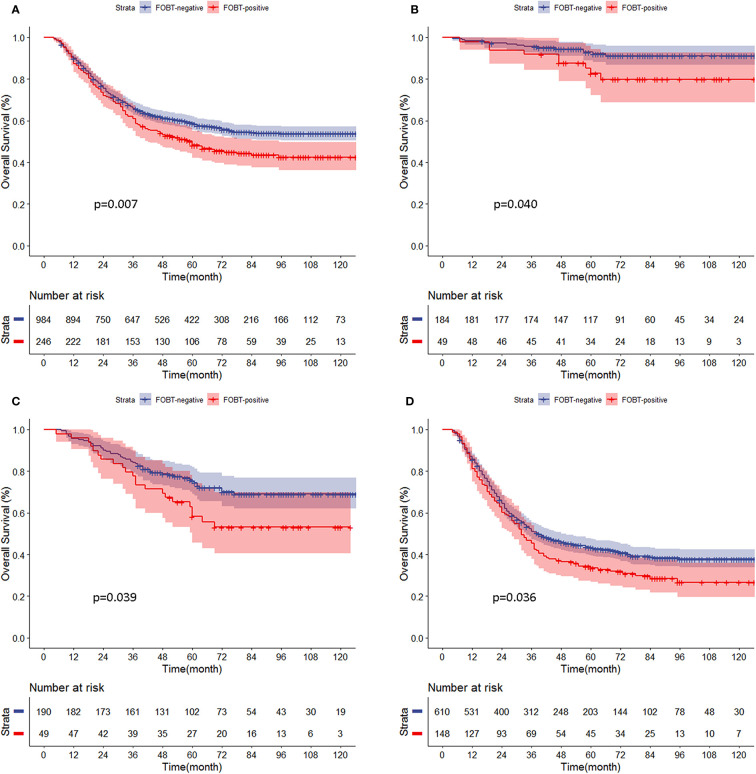
**(A–D)** Comparison of overall survival curves for propensity score-matched patients between the fecal occult blood test (FOBT)-positive and FOBT-negative groups according to pathological stage. **(A)** All patients. **(B)** p stage (I). **(C)** p stage (II). **(D)** p stage III.

Kaplan-Meier curve analysis showed that the prognosis of the FOBT-positive group was significantly worse than that of the FOBT-negative group (5-year OS: 48.2 *vs*. 58.8%, respectively, p = 0.007). Stratified analysis showed that the OS rate of the FOBT-positive group was significantly lower than that of the FOBT-negative group at all pathological stages (5-year OS: p stage I: 82.7 *vs*. 92.1%, p = 0.040; p stage II: 58.2 *vs*. 75.3%, p = 0.039; p stage III: 33.5 *vs*. 43.3%, p = 0.036) (**Figures 1A–D**). The same survival results were observed in the early GC group, advanced GC group, lymph node-negative group and lymph node-positive group (**Supplementary Figures S5A–D**). In addition, in a separate analysis of each clinicopathological factor, the prognostic value of the FOBT result was consistent, including age, tumor site, histologic, and so on (**Supplementary Figure S6**).

### Effects of FOBT Results on Adjuvant Chemotherapy

**Figure 2:**
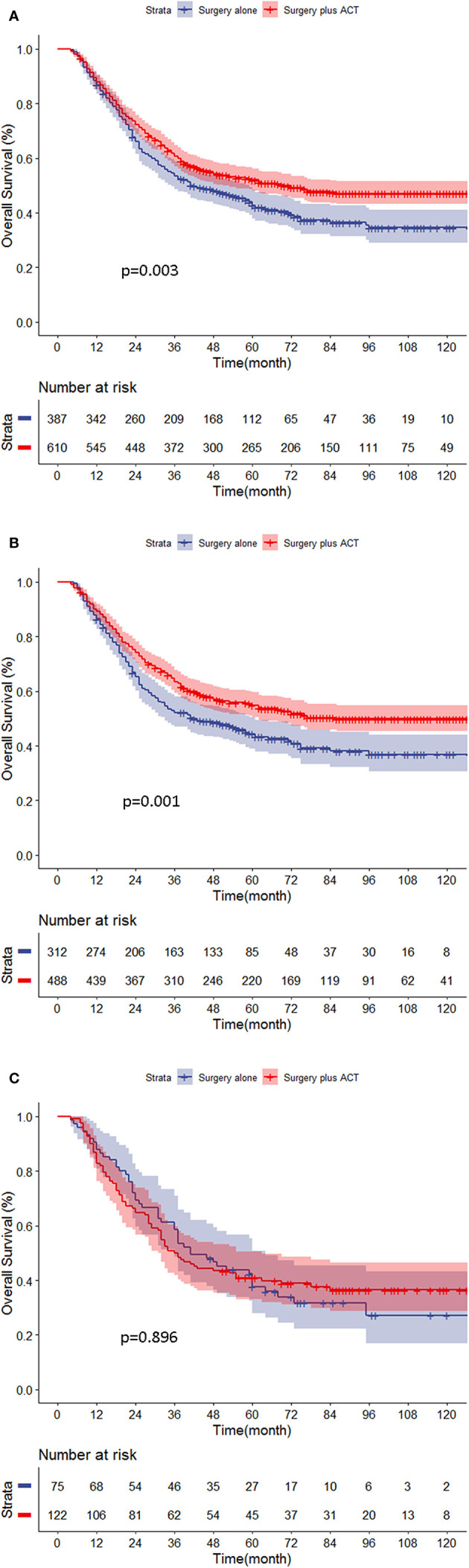
**(A–C)** Chemotherapy benefit analysis for stage II/III gastric cancer (GC) patients in different groups. **(A)** All stage II/III patients. **(B)** FOBT-negative patients. **(C)** FOBT-positive patients.

Further PAC benefit analysis for stage II/III GC patients after PSM showed that PAC improved the prognosis significantly (5-year OS: 52.1 *vs*. 42.8%, p = 0.003, respectively). Subgroup analysis showed that FOBT-negative patients benefited from PAC (5-year OS: 49.9 *vs*. 36.8%, p = 0.001). However, in the FOBT-positive patients, the prognosis was similar between the chemotherapy group and the non-chemotherapy group (5-year OS: 40.8 *vs*. 37.7%, respectively, p = 0.896) (**Figures 2A–C**).

### Immunohistochemistry Results of CD68, IL-6, and TNF-α Expression in Tumors

Paraffin-embedded sections of FOBT-negative and FOBT-positive patients (60 cases each) were randomly selected from the propensity score-matched patients (n = 1,230) for IHC analysis to explore the association between FOBT status and the tumor immune microenvironment (CD68, IL-6, and TNF-α expression). The clinicopathological characteristics between the two groups were comparable, showed in **Supplementary Table S1**. The CD68, IL-6, and TNF-α expression in tumor cells was significantly higher in FOBT-positive patients than in FOBT-negative patients (all p<0.05) (**Table 4**).

**Table 4:** Associations among fecal occult blood test (FOBT) result, CD68, IL-6, and TNF-α expression in tumor cells.

	FOBT (−) (n = 60)	FOBT (+) (n = 60)	p value	TNF-α low expression (n = 82)	TNF-α high expression (n = 38)	p value	IL-6 low expression (n = 70)	IL-6 high expression (n = 50)	p value
CD68				<0.001			<0.001		<0.001
Low expression	52 (86.7%)	34 (56.7%)		70 (85.4%)	16 (42.1%)		66 (94.3%)	20 (40.0%)	
High expression	8 (13.3%)	26 (43.3%)		12 (14.6%)	22 (57.9%)		4 (5.7%)	30 (60.0%)	
IL-6			0.010				<0.001		NA
Low expression	42 (70.0%)	28 (46.7%)		60 (73.2%)	10 (26.3%)		NA	NA	
High expression	18 (30.0%)	32 (53.3%)		22 (26.8%)	28 (73.7%)		NA	NA	
TNF-α				<0.001		NA			NA
Low expression	52 (86.7%)	30 (50.0%)		NA	NA		NA	NA	
High expression	8 (13.3%)	30 (50.0%)		NA	NA		NA	NA	

FOBT indicates fecal occult blood test; TNF-α, tumor necrosis factor-α; IL-6, interleukin-6.

## Discussion

In a study of individuals in the Netherlands undergoing screening for colorectal cancer by FOBT, it was found that fewer than 1% of patients with a positive result from the FOBT to receive a diagnosis of gastric cancers within 3 years (22). In the present study, we found that approximately 8% of GC patients had a preoperative FOBT-positive status, and FOBT-positivity in patients was associated with a worse clinical background and long-term prognosis. After further analyses with a 1:4 PSM, we found that patients with FOBT-positive tumors had a higher incidence of postoperative complications and a worse long-term prognosis than patients with FOBT-negative tumors. The chemotherapy benefit analysis of stage II/III GC patients found that FOBT-positive patients showed significant chemotherapy resistance. Finally, IHC analysis of tumor tissues in 120 patients showed that the TAM and the expression levels of IL-6 and TNF-α in tumor cells of FOBT-positive patients were significantly higher than those in FOBT-negative patients.

FOBT-positivity is common in digestive tract tumors (gastric cancer, colorectal cancer, etc.), inflammatory bowel disease, peptic ulcers and other diseases (23, 24). At present, the FOBT has been widely used in the screening of colorectal cancer in the general population (8). In recent years, studies by Libby and Chen et al. concerning large populations have shown that not only colorectal cancer mortality but also all-cause mortality and non-colorectal cancer mortality are significantly higher in patients who are FOBT-positive than in patients who are FOBT-negative (9, 10). It is speculated that the cause for this difference may be related to long-term chronic anemia (25, 26) and systemic inflammatory response (9), suggesting that the FOBT results can be a potential predictor of prognosis in a population. However, the relationship between FOBT results and long-term prognosis after radical surgery in GC patients has yet been reported.

Studies have shown that preoperative high CCI (27), low Hb level (28), and low albumin level (29) are associated with a worse prognosis. The present study also found that FOBT-positive patients had worse clinical backgrounds, including higher preoperative CCI, lower Hb, and Alb than FOBT-negative patients. After PSM, although the two groups had a similar baseline, the prognosis of the FOBT-positive group was significantly worse than that of the FOBT-negative group, and multivariate analysis showed that FOBT-positivity was an independent risk factor for worse prognosis. Therefore, we hypothesized that the mechanism may be related to TIM, postoperative complications, adjuvant chemotherapy resistance, and an imbalance of intestinal microbiota.

Since Virchow first discovered the relationship between inflammation and cancer (30), increasingly more evidence has shown that tumor progression is related not only to the intrinsic properties of the tumor cells but also to the local TIM (2). Tumor cells that highly express TNF-α stimulate M1-type macrophage infiltration and secrete IL-6, which damages mucosal epithelial cells and further causes recessive hemorrhage (14, 31–33). By IHC analysis, we demonstrated that the expression levels of CD68, IL-6 and TNF-α in tumor cells of FOBT-positive patients were significantly higher than those in FOBT-negative patients. This may explain why patients with positive FOBT had a more advanced tumor and worse prognosis.

In addition, more postoperative overall complications and a high incidence of anastomotic leakage in FOBT-positive patients may also affect prognosis. Similar to the results of our study, previous studies have shown that the occurrence of postoperative complications is closely related to the poor prognosis of GC patients, especially anastomotic leakage (34, 35). The mechanism may be related the presence of more significant postoperative inflammation and more severe immunosuppression in GC patients. Infectious complications and sepsis enhance the proinflammatory cytokine cascade, including TNF-α, IL-1, IL-6, and IL-8. These immunomodulators affect the function and regulation of natural killer cells, cytotoxic T lymphocytes, and antigen presenting cells (30, 36, 37). Moreover, postoperative complications can lead to prolonged immunosuppression, which enables residual tumor cells to proliferate and survive in the host for a longer time and promotes the occurrence of micrometastasis (34), which in turn affects the long-term prognosis. In addition, although the cause of postoperative complications associated with FOBT-positivity is not clear, based on the findings of our study, caution should be taken with regard to the potential for postoperative complications in FOBT-positive patients.

Postoperative adjuvant chemotherapy resistance may also affect the long-term prognosis of FOBT-positive GC patients. A large number of studies have confirmed that PAC can significantly improve the prognosis of stage II/III GC patients (38). However, it is also important to identify patients who will clearly benefit from chemotherapy. Zeng and Jiang found that the immune prognosis score based on TIM is related to the efficacy of PAC in GC patients and speculated that infiltration of lymphocytes in tumor cells indicates a chemotherapy-sensitive phenotype (2, 3). In our study, the K-M curves showed that although PAC significantly prolonged OS in the stage II/III group, but stratified analysis showed that FOBT-positive patients did not benefit from PAC. In addition, compared with the chromosomal instability subtypes found by Sohn et al. (4), the single patient classifier constructed by Cheong (5), and the microsatellite instability found by Ji and Young (6, 7), the FOBT has a high clinical use rate, is inexpensive and is more accessible. Although the mechanism underlying how FOBT-positivity induces chemotherapy resistance is still unclear, previous studies have shown that TAM and high expression of proinflammatory factors such as TNF-α and IL-6 are closely related to poor prognosis and chemoresistance (2, 3, 32, 39–41). In the present study, according to the results of the IHC analysis, we speculated that in patients with FOBT-positivity, local TIM (such as TAM, TNF-α, IL-6) is conducive to tumor proliferation and vascularization and is associated with adjuvant chemotherapy resistance. Therefore, to further improve the prognosis of FOBT-positive patients, it is necessary to carefully evaluate the potential applications of different adjuvant therapies and immune checkpoint therapy (40) in a well-designed, large-scale clinical trial for this particular subgroup of patients.

In addition, a study by Elinav has shown that systemic inflammation can alter the intestinal microbial composition, induce the amplification of genotoxic microorganisms and promote the development of digestive tract tumors (42). Intestinal microbiota can also affect local and systemic inflammation and play an important role in tumor treatment response (43, 44). Lida believed that the destruction of intestinal microbiota can reduce the treatment response of subcutaneous tumors in mice to chemotherapy drugs (43). Furthermore, intestinal microflora may also contribute to the formation of anticancer immune responses. The destruction of intestinal microflora can indirectly damage mucosal epithelial cells and negatively impact the prognosis of tumor patients (44). In addition, Japanese scholars found that an increase in the hemoglobin index of gastric mucosa is closely related to Helicobacter pylori (Hp) infection and mucosal inflammatory cell infiltration (45) and is more likely to cause gastric mucosal hemorrhage and FOBT-positivity. It is still unclear whether the occurrence of FOBT-positivity in GC patients and poor prognosis are related to the mucosal damage caused by intestinal bacterial flora imbalance, Hp infection or inflammatory cell infiltration, and further research is needed in the future to explore its relevance and the corresponding treatment measures (46).

There are many diseases that affect the results of FOBT, such as coronary heart disease, atrial fibrillation, due to a long-term use of anti-coagulation drugs. Intestinal bleeding may also cause by inflammatory bowel disease. However, we found that patients with these diseases only occupied a smaller proportion in the present cohort, and it may have limited influence on the results. In addition, in our center, most patients were diagnosed with GC by gastroscopy biopsy preoperatively, which may make the FOBT positive easily. After a definitive diagnosis, they underwent surgery within a week. No other intervention was applied unless they suffered several symptoms such as stomachache, belch, and so on. Due to the nature of the retrospective study, it’s difficult for us to further analyze the relationship between the treatment of these diseases and the results of FOBT. However, the hypothesis you propose is interesting and worth exploring in future research.

The present study has the following limitations: first, it is a single-center retrospective study with a certain bias and without external validation. Second, there were 2,124 patients with stage II/III GC, 1,164 (54.8%) of which received adjuvant chemotherapy. The proportion of patients with adjuvant chemotherapy in the present study was not high but was similar with previous study (4, 47, 48). Indeed, it’s difficult that all the target patient received adjuvant chemotherapy, which is a limitation and have a certain impact on our study. And we did not analyze the impact of adjuvant chemotherapy cycles on long-term survival. Third, we did not analyze the relationship between the FOBT results and recurrence patterns. Fourth, as an unresolved issue nowadays (2, 3), these specimens as well as semi-quantitative IHC evaluations may still not completely reflecting the tumor immune microenvironmental status. Last, because colonoscopy was not routinely performed in our department, we cannot exclude the patients with some intestinal diseases such as inflammatory bowel disease, which had the effect to the FOBT results. Nevertheless, for the first time, the present study used bulk data to report the relationship between FOBT results and the short- and long-term outcomes of GC patients after radical surgery and the efficacy of PAC in patients with stage II/III disease. Importantly, we used IHC analysis to speculate on the underlying mechanism. The findings will help clinicians develop the best treatment for FOBT-positive patients and provide direction for further research. Based on the current findings, more attention should be paid to FOBT-positive patients before, during, and after surgery. At the first visit, surgeons should determine if the FOBT-positive patient has any unfavorable clinical background (such as anemia and hypoalbuminemia) that may adversely affect the short-term outcome after gastrectomy and should take appropriate measures to reduce the incidence of early postoperative complications. During follow-up periods, the follow-up should be intensive, and because PAC has no clear effect, combined PAC with immunotherapy should be considered to improve the prognosis of these patients (39, 40, 46).

In summary, although the FOBT has been widely used clinically, its potential prognostic value for cancer patients is rarely reported. The present study demonstrated for the first time that FOBT results have predictive value for postoperative complications and long-term prognosis of GC. In addition, for stage II/III GC patients with FOBT-positive tumors, no significant benefit from PAC alone was observed. The role of FOBT and TAM in chemo-resistance should be explored and further evaluation of macrophage-targeting therapy is warranted. The present study can be used as background data for potential future large-scale, multicenter clinical trials to determine the best treatment decisions for these GC patients after surgery.

## Data Availability Statement

The data sets generated for this study are available on request to the corresponding authors.

## Ethics Statement

The studies involving human participants were reviewed and approved by the Institutional Review Board of Fujian Medical University Union Hospital. The patients/participants provided their written informed consent to participate in this study.

## Author Contributions

JL, BX, YX, CZ, and CH conceived of the study, analyzed the data, and drafted the manuscript. YX and YW evaluated the individual specimens. CZ, CH, and PL helped critically revise the manuscript for important intellectual content. PL, JX, JW, JXL, QC, and LC helped collect data and design the study. All authors contributed to the article and approved the submitted version.

## Funding

This study was funded by the National Nature Science Foundation of China (no. 81871899), Construction Project of Fujian Province Minimally Invasive Medical Center [no. (2017)171], Natural Science Foundation of Fujian Province (2019J01155), and Fujian provincial science and technology innovation joint fund project plan (2018Y9005) and Middle-aged and young backbone talent training project of Fujian Provincial Health Commission (2020GGA038).

## Conflict of Interest

The authors declare that the research was conducted in the absence of any commercial or financial relationships that could be construed as a potential conflict of interest.

The handling Editor declared a past co-authorship with several of the authors JX, CZ, CH, PL.

## Supplementary Material

Supplementary Figure 1 Flowchart depicting the patient selection process.

Supplementary Figure 2 Representative pictures of immunohistochemistry (magnification, ×200) which showed the different expression grades of macrophage infiltration in tumor cells.

Supplementary Figure 3 Representative pictures of immunohistochemistry (magnification, ×200) which showed the different expression grades of IL-6 infiltration in tumor cells.

Supplementary Figure 4 Representative pictures of immunohistochemistry (magnification, ×200) which showed the different expression grades of TNF-α infiltration in tumor cells.

Supplementary Figure 5 Comparison of overall survival curves for patients between the FOBT-positive and FOBT-negative groups according to pT and pN stage. **(A)** patients with pT1; **(B)** patients with pT2-4. **(C)** patients with pN0. **(D)** patients with pN+.

Supplementary Figure 6 The relationship between FOBT results and overall survival in various subgroups.
