# Supplementary figures and images for: A Novel Insight Into Fecal Occult Blood Test for the Management of Gastric Cancer: Complication, Survival, and Chemotherapy Benefit After R0 Resection
Source: Front Oncol. 2021 Feb 11;10:526746. doi: 10.3389/fonc.2020.526746 (PMC7905191; doi:10.3389/fonc.2020.526746)

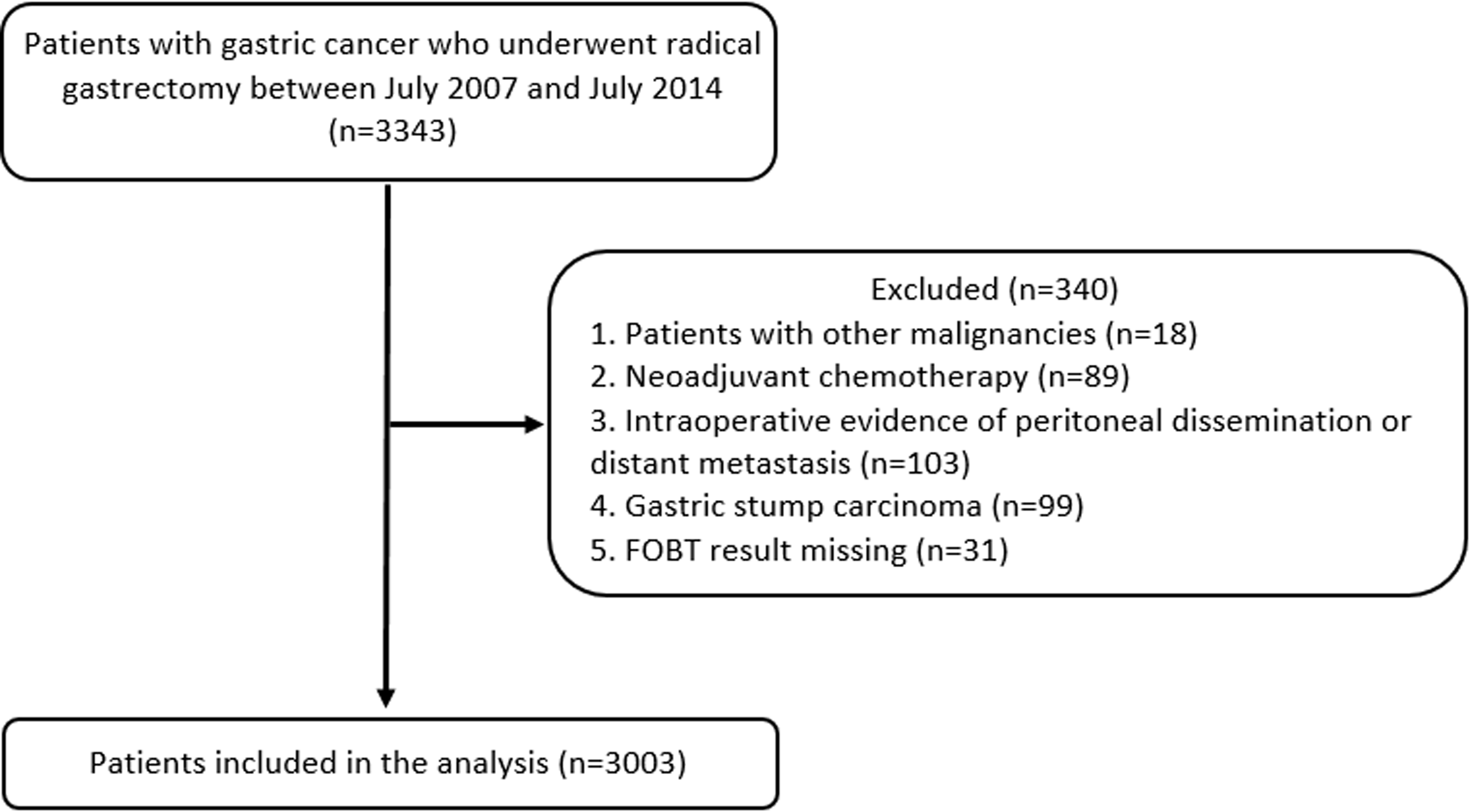

Supplement: Supplementary Figure 1 — Flowchart depicting the patient selection process. [file Image_1.tif]

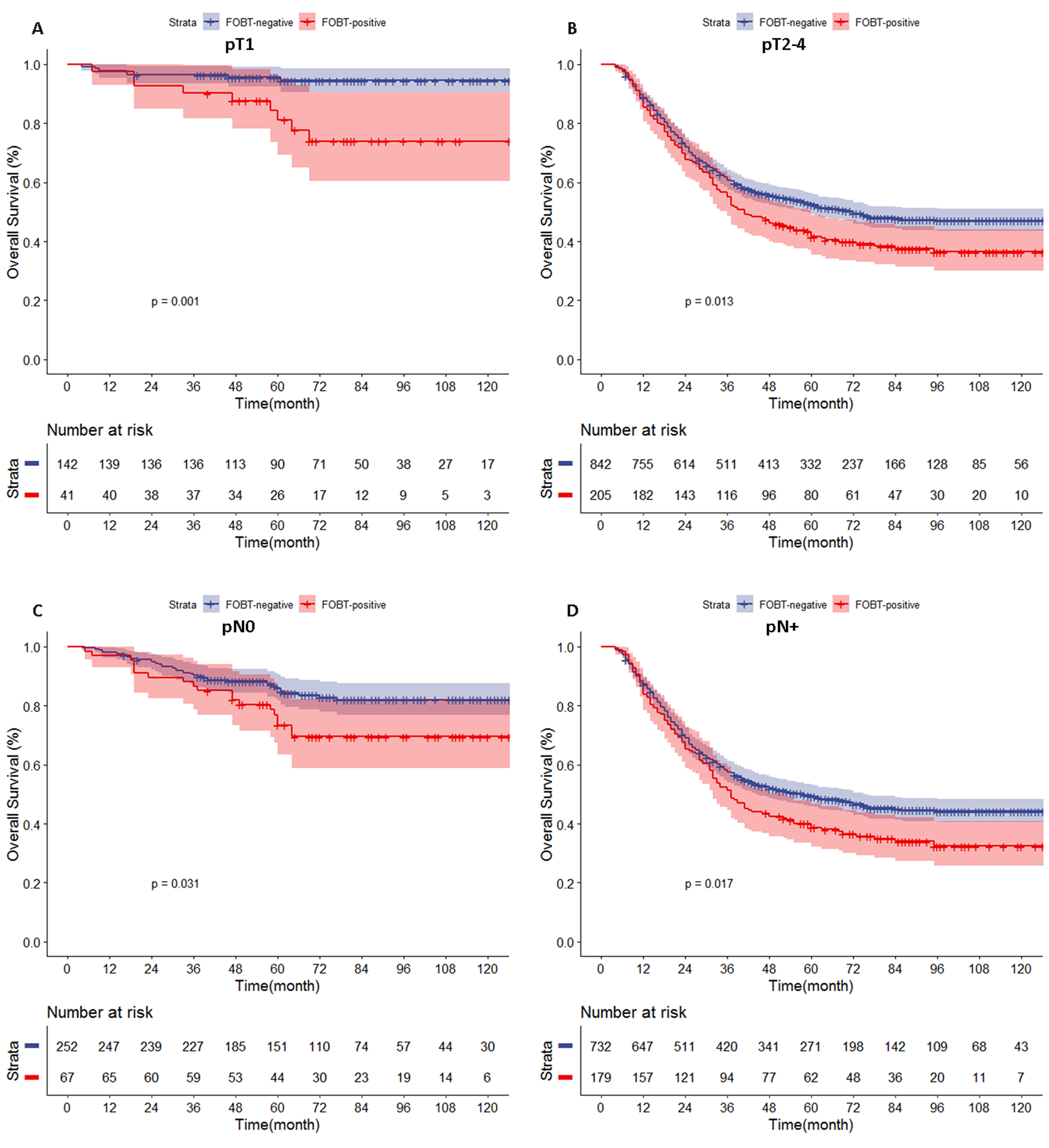

Supplement: Supplementary Figure 5 — Comparison of overall survival curves for patients between the FOBT-positive and FOBT-negative groups according to pT and pN stage. (A) patients with pT1; (B) patients with pT2-4. (C) patients with pN0. (D) patients with pN+. [file Image_5.tif]

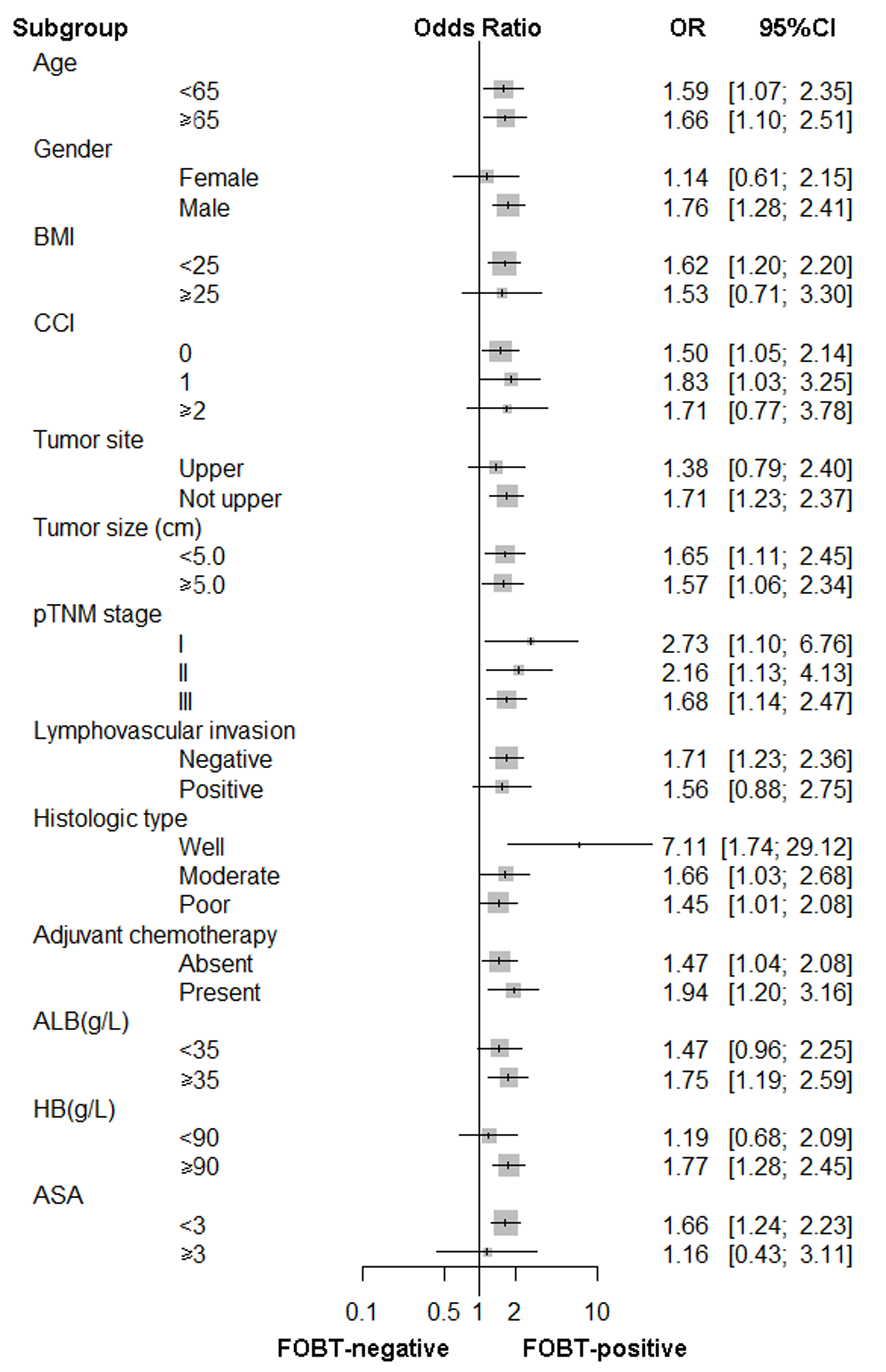

Supplement: Supplementary Figure 6 — The relationship between FOBT results and overall survival in various subgroups. [file Image_6.tif]
