# Supplementary material for: A Novel Insight Into Fecal Occult Blood Test for the Management of Gastric Cancer: Complication, Survival, and Chemotherapy Benefit After R0 Resection
Source: Front Oncol. 2021 Feb 11;10:526746. doi: 10.3389/fonc.2020.526746 (PMC7905191; doi:10.3389/fonc.2020.526746)
Supplement: Supplementary file 7 [file Table_1.docx]

| **Table S1 Clinicopathological characteristics of patients selected for IHC.** | | | |
| --- | --- | --- | --- |
|  | Patients selected for IHC | | |
|  | FOBT (-) (n=60) | FOBT (+) (n=60) | p value |
| Age |  |  | 0.307 |
| <65 | 36 (60.0%) | 33 (55.0%) |  |
| ≥65 | 24 (40.0%) | 27 (45.0%) |  |
| Sex n (%) |  |  | 0.526 |
| Female | 12 (20.0%) | 12 (20.0%) |  |
| Male | 48 (80.0%) | 48 (80.0%) |  |
| Charlson comorbidity index, n (%) |  |  | 0.600 |
| 0 | 38 (63.3%) | 34 (56.7%) |  |
| 1 | 14 (23.3%) | 14 (23.3%) |  |
| ≥2 | 8 (13.3%) | 12 (20.0%) |  |
| ASA |  |  | 0.769 |
| <3 | 54 (90.0%) | 53 (88.3%) |  |
| ≥3 | 6 (10.0%) | 7 (11.7%) |  |
| BMI |  |  | 1.000 |
| <25 | 52 (86.7%) | 52 (86.7%) |  |
| ≥25 | 8 (13.3%) | 8 (13.3%) |  |
| Tumor size n (%) |  |  | 0.583 |
| <50 mm | 29 (48.3%) | 26 (43.3%) |  |
| ≥50 mm | 31 (51.7%) | 34 (56.7%) |  |
| Tumor location n (%) |  |  | 0.797 |
| Upper | 17 (28.3%) | 19 (31.7%) |  |
| Middle | 12 (20.0%) | 13 (21.7%) |  |
| Lower | 26 (43.3%) | 21 (35.0%) |  |
| Mix | 5 (8.3%) | 7 (11.7%) |  |
| Histologic type n (%) |  |  | 0.711 |
| Differentiated | 24 (40.0%) | 26 (43.3%) |  |
| Undifferentiated | 36 (60.0%) | 34 (56.7%) |  |
| Lymphovascular invasion n (%) |  |  | 0.648 |
| Absent | 47 (78.3%) | 49 (81.7%) |  |
| Present | 13 (21.7%) | 11 (18.3%) |  |
| Adjuvant chemotherapy n (%) |  |  | 0.855 |
| Absent | 28 (46.7%) | 27 (45.0%) |  |
| Present | 32 (53.3%) | 33 (55.0%) |  |
| pTNM stage n (%) |  |  | 0.289 |
| Ⅰ | 9 (15.0%) | 16 (26.7%) |  |
| Ⅱ | 9 (15.0%) | 8 (13.3%) |  |
| Ⅲ | 42 (70.0%) | 36 (60.0%) |  |
| Hemoglobin n (%) |  |  | 1.000 |
| <90 g/L | 13 (21.7%) | 13 (21.7%) |  |
| ≥90 g/L | 47 (78.3%) | 47 (78.3%) |  |
| Albumin n (%) |  |  | 0.529 |
| <35 g/L | 26 (43.3%) | 31(51.7%) |  |
| ≥35 g/L | 34 (56.7%) | 29 (48.3%) |  |
| FOBT indicates faecal occult blood test; ASA, American Society of Anesthesiologists; BMI, body mass index | | | |
